# A Common Medication with an Uncommon Adverse Event: A Case of Doxycycline-induced Pancreatitis

**DOI:** 10.7759/cureus.7496

**Published:** 2020-04-01

**Authors:** Shweta Paulraj, Prashanth Ashok Kumar, Dinesh Subedi

**Affiliations:** 1 Internal Medicine, Upstate Medical University, Syracuse, USA

**Keywords:** drug-induced pancreatitis, doxycycline, acute pancreatitis

## Abstract

Drug-induced pancreatitis is a rare entity. The diagnostic criteria for drug-induced pancreatitis include the development of pancreatitis during drug therapy, elimination of all other possible causes, resolution with discontinuation of the offending drug, and reappearance on using the same drug. Several drugs have been implicated in having an association with pancreatitis. Tetracyclines are considered to be a Class I medication (medications implicated in greater than 20 reported cases of acute pancreatitis). However, there are very few reported cases of doxycycline-induced acute pancreatitis. We report the case of a 55-year old male who presented to the emergency department (ED) with three days of progressively severe and constant mid-epigastric abdominal pain. On evaluation, he was found to have elevated lipase levels. Computed tomography (CT) scan of his abdomen revealed findings consistent with pancreatitis without any evidence of gallstones or common bile duct dilation. He denied alcohol use, trauma, and insect bites or stings. His calcium and triglyceride levels were within normal limits. His blood cultures did not show any bacterial growth. He had recently been initiated on doxycycline for concerns of cellulitis and had begun to develop abdominal pain seven days after the initiation of doxycycline. He had completed his antibiotic course on the day of presentation to the ED. He had no other recent medication changes. He had subsequent improvement of symptoms off of the doxycycline and with supportive care. Given that all other causes of pancreatitis had been excluded and that he had been initiated on doxycycline prior to presentation, the etiology was attributed to being likely secondary to doxycycline use. Our case highlights the importance of reviewing outpatient medications by the hospital medicine team and awareness of rare triggers for acute pancreatitis.

## Introduction

Acute pancreatitis is a common cause of hospitalization amongst gastrointestinal disorders in the United States (US). There are a multitude of conditions that can result in acute pancreatitis, most commonly, alcohol use, gallstones, and hypertriglyceridemia [1]. Of the numerous other causes of acute pancreatitis, drug-induced pancreatitis is a rare entity with an incidence ranging from 1.4% - 5.3%, according to various meta-analyses and studies [2-4]. Several drugs have been implicated in having an association with pancreatitis. However, there are very few reported cases of doxycycline-induced acute pancreatitis. We report the rare case of a patient presenting with acute pancreatitis following treatment with doxycycline.

## Case presentation

A 55-year-old male with a past medical history of hypertension, diabetes mellitus type 2, gastroesophageal reflux disease, and quadriplegia from trauma eight years prior presented to the emergency department (ED) with three days of progressively severe and constant mid-epigastric abdominal pain. His pain was associated with eating, rated 9/10, and persistent even at rest. He had also been having decreased appetite, nausea, and diarrhea for three days. He did not have a history of alcohol use. He did not have any recent trauma, insect bites, or stings. He had undergone flap reconstruction for a Stage 4 pressure ulcer a few months back. He had just completed a 10-day course of doxycycline for concerns for flap site cellulitis prior to presentation and had begun to develop abdominal pain since the seventh day of doxycycline use. He also reported chronic use of marijuana and had been on fenofibrate, duloxetine, trazodone, and metformin for several years.

**Figure 1:**
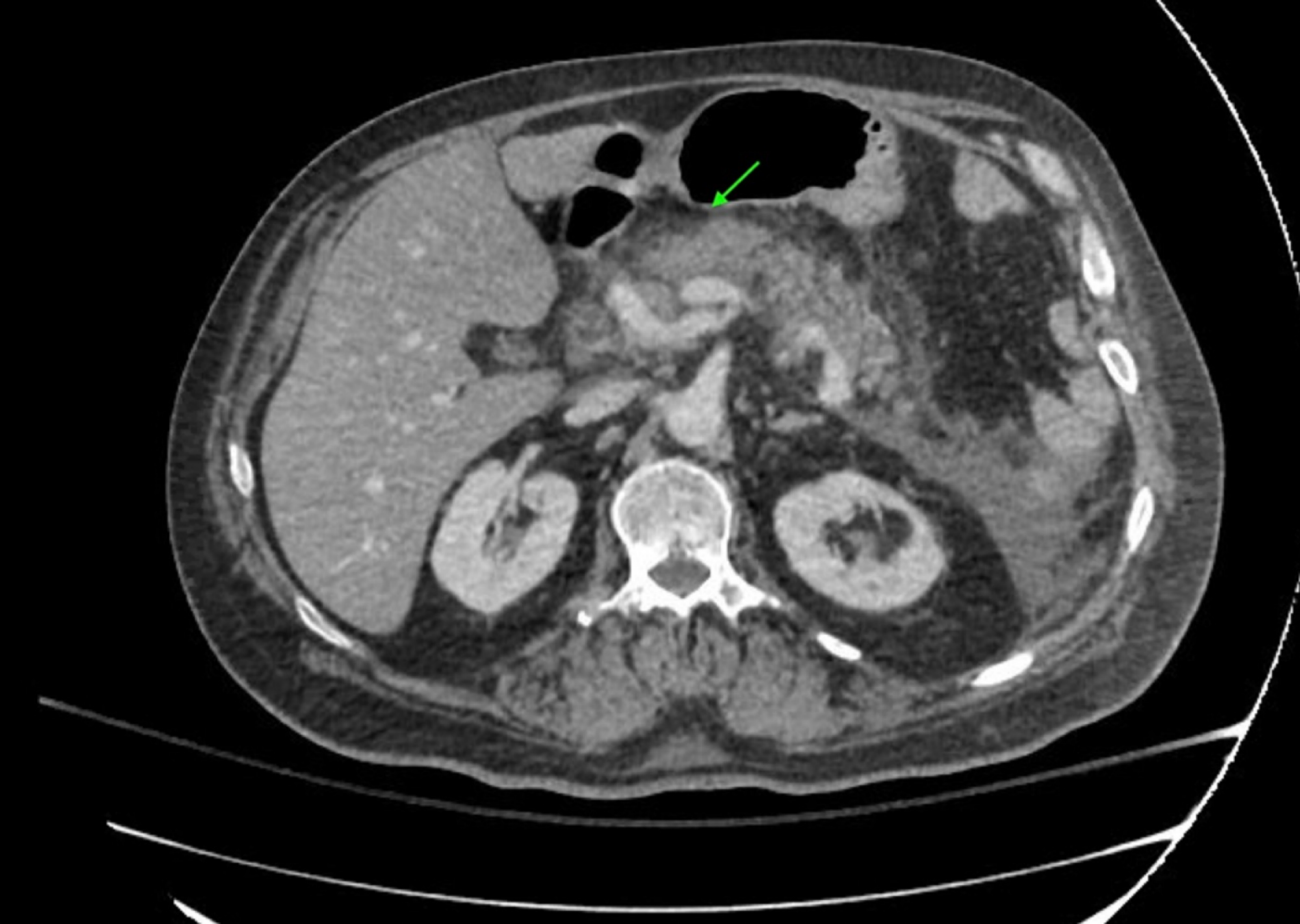
Computed tomography (CT) finding of acute pancreatitis Arrow depicts peripancreatic edema.

On initial examination, he was tachycardic and tachypneic with abdominal distention and tenderness. Initial laboratory evaluation was remarkable for leukocytosis with an elevated lipase at 2,726 U/L (normal: < 95 U/L). His triglyceride and calcium levels were within normal limits. His lactic acid levels were also elevated with a high anion gap. Computed tomography (CT) scan of his abdomen revealed moderate peripancreatic edema extending along the retroperitoneal fascia into both pericolic gutters (Figure 1). There was no evidence of gall stones or bile duct dilation. Blood cultures were also obtained due to his leukocytosis and history of recent cellulitis but did not show any bacterial growth. Stool testing was negative for Clostridium difficile.

He was treated with aggressive fluid hydration, bowel rest, and careful monitoring. His home medications of fenofibrate, trazodone, duloxetine, and metformin were held on admission. Duloxetine and trazodone were reintroduced during the course of his hospital stay with no worsening of symptoms. His diet was gradually advanced. He had significant improvement in his symptoms through his stay and had returned to baseline functioning with supportive care. His fenofibrate was resumed on discharge. Given that all other causes of pancreatitis had been excluded and that he had been initiated on doxycycline prior to presentation, the etiology was attributed to being likely secondary to doxycycline use.

## Discussion

Doxycycline is a commonly used antibiotic and has been effectively used in a wide array of infectious and inflammatory diseases. The commonly reported adverse effects of doxycycline include gastrointestinal symptoms like diarrhea, esophagitis, rare cases of hepatotoxicity, photosensitivity, and hypersensitivity reactions. Doxycycline-induced pancreatitis has been reported in less than 1% of patients who take doxycycline [5].

The diagnostic criteria for drug-induced pancreatitis include the development of pancreatitis during drug therapy, elimination of all other possible causes, resolution with discontinuation of the offending drug, and reappearance of symptoms on using the same drug [6]. Tetracyclines are considered to be a Class I medication (medications implicated in greater than 20 reported cases of acute pancreatitis) [7-8]. There are only a handful of cases reported of doxycycline-induced pancreatitis per a literature review. Of the reported cases of doxycycline-induced pancreatitis, it has been reported more commonly in females [2-4].

There are no clinical symptoms, signs, or laboratory criteria specific for drug-induced pancreatitis. The time to onset of doxycycline-induced pancreatitis per prior case reports has been variable as shown in Table 1. Other demographic variables have also been depicted in Table 1.

**Table 1:** Comparison of Reported Cases of Doxycycline-induced Pancreatitis NR: not reported

	Case 1	Case 2	Case 3	Case 4	Case 5	Case 6	Current case
Age	58	52	21	33	75	51	55
Gender	female	female	female	female	female	male	male
Dose of doxycycline	400 mg/day	200 mg/day	NR	1,000 mg/day	400 mg/day	200 mg/day	200 mg/day
Time to symptoms	2 days	7 days	15 days	3 days	14 days	3 days	7 days
Lipase levels (U/L)	2,508	> 1,600	128	595	NR	5,485	2,726
Other potential medications	NR	Gabapentin, oxycodone. No recurrence with rechallenge of these medications	NR	Ornidazole	NR	none	Fenofibrate for 10 years, chronic marijuana use
Reference number:	Inayat et al. [9]	Rawla et al. [10]	Wachira et al. [11]	Ocal et al. [12]	Achecar Justo et al. [13]	Moy et al. [14]	Current case

As evident from the table, there seems to be a wide variation in the population of patients affected with a female predominance. Given the rarity of the condition, doxycycline-induced pancreatitis has been believed to be an idiosyncratic reaction. Other postulated mechanisms for doxycycline-induced pancreatitis include cytotoxic and metabolic effects, accumulation of a toxic metabolite or intermediary, hypersensitivity, and idiosyncratic reactions [9, 15].

In our patient, another potential etiology for pancreatitis could have been fenofibrate, which was also held on admission. However, he had been on the medication for over a year and doxycycline was the only new medication that he had been taking prior to presentation. Regardless, both the medications were held on the presentation.

## Conclusions

Doxycycline is a very commonly used antibiotic that can be an uncommon trigger for acute pancreatitis. Our case aims to propagate awareness of such uncommon causes of acute pancreatitis. It also highlights the need for thorough history taking and outpatient medication review on admission. This will enable early identification and discontinuation of the offending drug in patients with a high clinical suspicion for the same.
